# Integrating China in the International Consortium for Personalised Medicine: a position paper on innovation and digitalization in Personalized Medicine

**DOI:** 10.1186/s12889-024-18009-8

**Published:** 2024-02-14

**Authors:** Flavia Beccia, Marzia Di Marcantonio, Francesco Andrea Causio, Lena Schleicher, Lili Wang, Chiara Cadeddu, Walter Ricciardi, Stefania Boccia

**Affiliations:** 1 Section of Hygiene, University Department of Life Sciences and Public Health, Università Cattolica del Sacro Cuore, Roma, Italia; 2 Faculty of Economics, Università Cattolica del Sacro Cuore, Rome, Italy; 3 Steinbeis Europa Zentrum (SEZ), Stuttgart, Germany; 4 BGI-Shenzhen, Shenzhen, CN China; 5 Department of Woman and Child Health and Public Health, Fondazione Policlinico Universitario A. Gemelli IRCCS, Roma, Italia

**Keywords:** European Union, China, Big Data, ICT solutions, Innovation, funding, Personalised medicine

## Abstract

**Background:**

The advent of Personalized Medicine (PM) holds significant promise in revolutionizing healthcare by tailoring treatments to individual patients based on their data. However, its successful implementation requires the seamless integration of innovative technologies and presents formidable challenges in terms of sustainability. To tackle these challenges head-on, the International Consortium for Personalized Medicine (ICPerMed) was established, and the IC2PerMed project, as part of this consortium, seeks to foster collaboration between the European Union (EU) and China in the field of Personalized Medicine. Based on the results collected by the project, the objective of this study is to discern the key priorities for the implementation of Personalised Medicine concerning Information and Communication Technologies (ICT) and Big Data and digital solutions, with a particular emphasis on data management and protection.

**Methods:**

A Delphi survey was conducted to gather expert’s consensus on the main priorities for actions on Information and Communication Technologies (ICT) and Big Data and digital solutions in the field of Personalized Medicine.

**Results:**

The survey identified seven priorities in the area of Big Data and digital solutions, including data interoperability, standards, security measures, and international partnerships. Additionally, twelve priorities were identified for the innovation-to-market process, emphasizing cost-effectiveness, need assessment, and value definition in resource allocation.

**Conclusions:**

The effective implementation of new technologies in Personalized Medicine research and practice is essential for the advancement of healthcare systems in both the European and Chinese contexts. The identified priorities play a pivotal role in promoting the sustainability of health systems and driving innovation in the implementation of Personalized Medicine. Addressing challenges related to data interoperability, standards, security, international collaboration, cost-effectiveness, and value assessment is of utmost importance in order to propel the progress of Personalized Medicine in healthcare systems.

## Background

Personalized Medicine (PM) has promised to revolutionize healthcare by tailoring prevention, diagnosis, and treatments through the collection of genotype and phenotype data adapted to specific individuals [1]. However, implementing PM requires that innovative technologies are adopted rather than rejected, which poses significant challenges regarding the sustainability of the process [2]. The implementation of Big Data and digital tools in health systems can improve their sustainability and have a positive impact on the quality of care, but the necessary transition to innovative digital tools is still far from being complete from the macro to the micro level, where their adoption is still fragmented [3, 4]. Involving stakeholders globally in defining shared research and development approaches, standards, and priorities should turn PM into an opportunity for all citizens and patients [5]. The study agenda of the European Commission (EC) places on these topics a high priority, facilitating in 2016 the creation of the International Consortium for Personalized Medicine (ICPerMed). The Consortium aims at developing financing initiatives and support measures, including the launch and funding of the initiative "Integrating China in the International Consortium for Personalized Medicine" (IC2PerMed). IC2PerMed is seeking to address all the above-mentioned issues by enabling the development of a shared strategy for PM research, innovation, development, and application between the EU and China [6, 7]. These efforts were coupled with the growing attention paid by China to digital health and innovation. In the 15-year plan called “Precision Medicine Initiatives”, China focused on the design of PM sector and envisioned the healthcare system reform by building it on new clinical life sciences technologies, large-scale cohort studies, Big Data platforms and infrastructure, and sustainability [8].

The present work, created as part of IC2PerMed project, seeks to identify, through a Delphi survey of experts, the priorities relating to the implementation of Information and Communication Technologies (ICT) and Big Data in the PM scenario, in EU and China.

## Methods

Three online workshops were organized as part of the IC2PerMed project to determine the priorities areas in PM. The choice of subjects addressed in the workshops is derived from an examination of the primary priorities outlined in the 'ICPerMed Vision for 2030,' in conjunction with the most significant findings from a comprehensive mapping exercise pertaining to PM policies and initiatives in both Europe and China. The extensive details of the mapping outcomes can be found in the project public deliverables, which are accessible on the IC2PerMed website, and related scientific papers [1, 3, 6, 7, 9, 10]. This manuscript presets the outcomes of one specific workshop, which delved into the essential guiding inquiries pertaining to the utilization of Big Data and ICT solutions for PM and the facilitation of innovation within the market to advance PM in both the European Union (EU) and China. A bottom-up and top-down strategy was used to identify the European and Chinese experts who would be invited in late 2020, and the result included 47 experts, of which 37 from the EU and 10 from China. The methodology for expert recruitment is reported in a specific deliverable of the project ("List of WG members and working procedures”) [11]. In summary, experts were identified through a top-down approach of identifying experts in the field through publications, partnerships, and projects, and a bottom-up approach through an open call for experts on the project website and open, partner-led dissemination and communication activities along project developments. Despite the initial appearance of this disparity as a potential concern, it has been determined that variations in response rates among different countries can be attributed to cultural factors and other relevant considerations, which do not substantially impact the collected results [12].

Prior to the workshop, participants were given a collection of guiding questions regarding the mapping exercise of European and Chinese policies in PM [13].

Following the identification of preliminary objectives, a two-round Delphi expert survey was conducted to reach consensus [14]. The survey consisted of 36 items, which were split into two sections and included 4 questions on demographic data, 15 questions on Big Data and ICT solutions, and 17 questions on bringing innovation to market. The survey was initially created in English and subsequently translated, also, into Chinese by an authorized translator to avoid potential language barriers or misinterpretations.

International experts with in-depth knowledge of the subject were asked to examine the items’ content and evaluate each one’s validity and applicability using a five-point Likert scale (1 being strongly disagreed with and 5 being strongly agreed with). Experts could offer extra priorities based on their experience or provide feedback on the priorities presented during the Delphi rounds, thanks to a final open-ended question. The poll was revised after the first round, incorporating comments from each participant, and sent back to the panel for the second round of consultation. Implementation of the Delphi took place between March and May of 2021. The entire process was carried out granting confidentiality, and participants were asked to disclose any potential conflicts of interest. Consensus was estimated in terms of Content Validity Index (CVI) [14, 15]. The CVI represents the proportion of experts who rate a single item with a score of 4 or 5 to the total number of experts engaged, and it ranges from 0 to 1, or from 0 to 100%. According to scientific literature, we determined that an agreement level of 80% was the threshold at which an item was deemed appropriate for inclusion; if it fell between 70 and 79%, it suggested a need for item revision; and if the CVI was less than 70%, it indicated potential item removal [9, 16–20]. This tool was used to outline the final set of priorities, not to suggest an order of importance among them. Therefore, all priorities included are to be considered equally important.

## Results

**Fig. 1:**
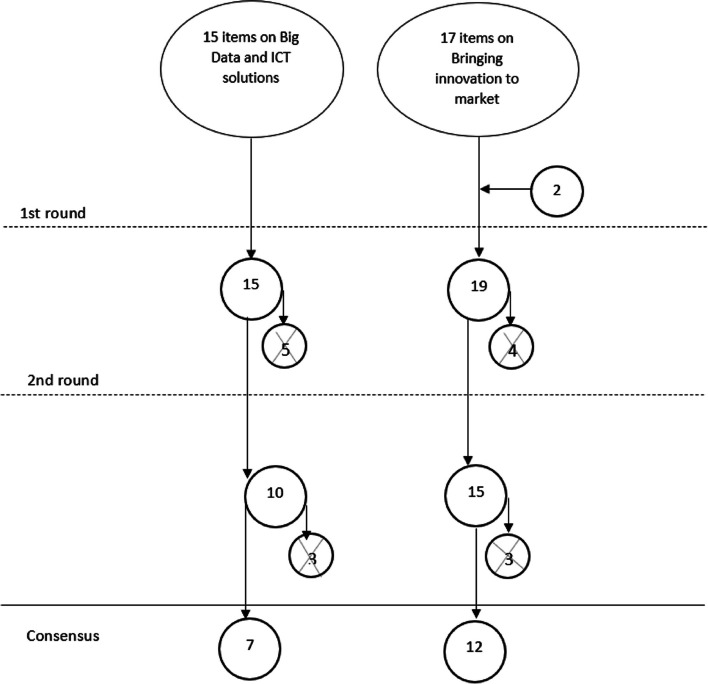
Delphi survey flowchart. The vertical flow indicates the number of priorities stemming before and after each Delphi survey round. The circles containing an X mark the number of priorities eliminated after each Delphi survey round. Horizontal arrows refer to priorities that were included following experts’ suggestions or any significant inputs from the workshops

Two topics were identified concerning innovation and market: 1) Big Data and ICT solutions; and 2) bringing innovation to market. The Delphi survey flowchart is detailed in Fig. 1 and the final priorities are listed in Table 1

**Table 1:** Final priorities in Big Data and ICT solutions and Bringing innovation to the market

*Topic*	*Priority*	
Big Data and ICT solutions	1	Select Big Data and make them analysable, comparable, and interoperable across borders
	2	Consider data security measures when developing new ICT solutions
	3	Enforce the implementation and usage of data standards in Personalised Medicine
	4	Adopt ICT solutions to health challenges globally, not just focusing on high-income countries
	5	Foster global efforts and joint policies for creating safe zones for cross-border data sharing
	6	Stimulate cooperation between academia and industry, facilitating data exchange procedures
	7	Embrace the societal and cultural differences among countries with the aim of safeguarding public trust in government and state authorities
Bringing innovation to the market	1	Ensure PM stakeholders follow shared principles and universal data sharing and exchange guidelines when using advanced diagnostics
	2	Focus newly marketed solutions on maximising patient health outcomes
	3	Assure that health insurance providers extend their coverage to innovative PM solutions and that the reimbursement of services is guaranteed
	4	Reduce the economic costs and lower the barriers to the market uptake of personalised diagnostics
	5	Intensify the dialogue between public health systems and the developers of PM solutions and products
	6	Urge stakeholders to have a holistic and long-term perspective on the budget when placing priorities on innovation
	7	Elaborate on cost-effectiveness and economic analysis models that consider the social and healthcare budgets
	8	Extend the role of personalised diagnostics in sustainable medical treatment
	9	There is a greater need for early, intense, coordinated, and continued crosstalk among all PM stakeholders to support breakthrough innovations
	10	Reward innovations that aim for a higher therapeutic value and guide innovation efforts towards the most relevant areas
	11	Develop methodologies to measure the social value of pharmaceutical products and systematically use such methods, e.g., in the context of health technology assessment
	12	Keep into account the interconnection and mutual dependency between diagnostics and therapeutics

The priorities identified during the Delphi survey process are hereby reported in the original topic where they were elaborated. They are reported together with a brief description to present them.

### Big data and ICT solutions

Fifteen priorities were identified in the preliminary research. Five priorities were removed after the first Delphi round and three after the second round. Seven priorities reached a consensus.

#### Priority 1: select big data and make them analysable, comparable, and interoperable across borders

With respect to the vast amount of data required to implement PM solutions and to the limited resources available to store them, there is the need to carefully identify the type of information to be retained, increasingly favouring those related to health outcomes rather than information with no proven clinical or management value. Data on biomarkers, genetic risk factors, disease outcomes, individual lifestyles, environmental factors, personal behaviour or medical treatment responses, all depend on the development of Big Data analytics that leads to similar outcomes, independently from the technological platform and algorithmic models applied, especially with regard to the integration of multivariate data structures. Since data accession is a common and complex problem to solve the entire data processing pipeline bottleneck, it is essential to study solutions to effectively combine data from different sources, focusing on their standardisation for practical usage.

#### Priority 2: consider data security measures when developing new ICT solutions

Handling health data requires strict security measures to ensure citizen ownership and privacy rights when deidentification techniques are not feasible or they lower the medical value for personalised approaches. Current data protection regulations should therefore be kept into consideration when developing new ICT solutions while also addressing potential future policies on data security measures. New ICT solutions should therefore be compatible with existing policy norms to ensure smooth adoption.

#### Priority 3: enforce the implementation and usage of data standards in Personalised Medicine

The amount of health data produced by everyday medical practice is increasing and promising for clinical and research purposes. However, the lack of data standards undermines the benefits that can derive from such data, limiting their use for secondary purposes and their exchangeability among different partners. The IC2PerMed expert panel concluded that data standardisation efforts in Europe and China are essential for stakeholders to benefit from increased data availability. In terms of standardisation, it is also necessary to identify responsible figures for data collection regarding PREMs (Patient Reported Experience Measures), PROMs (Patient Reported Outcome Measures) and CROMs (Clinician Reported Outcome Measures).

#### Priority 4: adopt ICT solutions to health challenges globally, not just focusing on high-income countries

The scarcity of financial resources might lead low- and middle-income countries to delay the adoption of ICT solutions or have them adopt different technological solutions than those in high-income countries. This heterogeneity could have adverse effects on data exchangeability and the development of good practices, among other issues: to ensure a truly global development of PM, global harmonisation of adopted ICT infrastructure is crucial.

#### Priority 5: foster global efforts and joint policies for creating safe zones for cross-border data sharing

Creating safe zones for a secure processing environment for protecting sensitive information remains a very interesting prospect with great potential for PM research purposes, both on the Chinese and the European sides. Both sides are working on "common health data spaces" to be launched soon, whose goal is to allow for safe sharing of health data, for primary and secondary purposes. Shared work and troubleshooting deriving from previous steps in implementing these projects could lead to benefits for both sides, avoiding effort duplication and reducing dead ends.

#### Priority 6: stimulate cooperation between academia and industry, facilitating data exchange procedures

Intensifying collaboration between academia and industry by means of dedicated workshop events, shared conferences, and similar, to elaborate strategies to use the data from electronic medical records, genomic, behavioural, and environmental data more efficiently is crucial for getting the most benefit out of them. This collaboration should bring together different forms of expertise and maximise value extraction from these data. To this purpose, the framework conditions for this collaboration must be improved and optimised to ensure that people working in different fields and with heterogeneous backgrounds can contribute at their best.

#### Priority 7: embrace the societal and cultural differences among countries with the aim of safeguarding public trust in government and state authorities

Public trust in government and state authorities differs significantly among countries and strongly depends on their socio-historical contexts. Both on the Chinese and European sides, societal and cultural differences are often seen as a hurdle for international collaboration on PM. A global approach to public health must embrace discrepancies and find ways to cooperate in Research and Innovation (R&I), tackling the cultural distance between Europe and China to ensure that collaboration and joint efforts don’t lead to the erosion of trust in government and state authorities, but rather serve as a catalyst for unanticipated opportunities.

### Bringing innovation to market

Starting from 17 preliminary priorities, four priorities were eliminated, two were added during the first round, and three additional ones were excluded in the second round. Twelve priorities reached a consensus.

#### Priority 1: ensure PM stakeholders follow shared principles and universal data sharing and exchange guidelines when using advanced diagnostics

Advances in Big Data and ICT solutions led to ground-breaking PM diagnostic innovations enabled by Artificial Intelligence (AI) models and machine learning. Such advanced diagnostics are highly dependent on health data available for elaboration, which is not always the case. Agreeing on a set of shared principles and universal guidelines on data sharing and exchange is crucial to ensure PM implementation in the healthcare system since the entire diagnostic process leans on the accessibility and availability of diverse and complex data sources.

#### Priority 2: focus newly marketed solutions on maximising patient health outcomes

The development of new solutions through translational clinical research is focussed on the regulatory approval process through national and international market authorisation agencies. However, for a new solution to be in the hands of patients and to be applied in the healthcare system, downstream processes in the market uptake value chain are equally important, such as Health Technology Assessment (HTA) and elaboration of reimbursement mechanisms, either by adaptation of existing ones or creation of new ones. Ensuring innovators’ focus is shifted on generating value and maximising patient health outcomes, rather than getting their solutions approved, would avoid the development of solutions of doubtful value.

#### Priority 3: assure that health insurance providers extend their coverage to innovative PM solutions and that the reimbursement of services is guaranteed

Innovative PM solutions, such as Advanced Therapy Medicinal Products (ATMP) in the context of gene- or cell-based therapies, are often associated with high costs for research, development, and production. Their adoption rate could rise by pushing an outcome-based reimbursement model on cost- and risk-sharing principles between the payer (e.g., health insurance) and the manufacturer. Having the manufacturers finance unsuccessful treatments themselves and receive reimbursement only upon patients responding to the therapeutic approach, potentially including success fees, would have a positive effect.

#### Priority 4: reduce the economic costs and lower the barriers to the market uptake of personalised diagnostics

The promise of PM that patient stratification procedures lower the overall burden on the healthcare budget is key to its market uptake. The experts of Working Group 2 have reached a consensus that personalised diagnostics should prove to be cost-efficient compared to conventional diagnostics. This would in turn have a positive effect lowering the barriers to their market uptake.

#### Priority 5: intensify the dialogue between public health systems and the developers of PM solutions and products

R&I funding is key to incentivising the development of products and services that serve the interests of public health. However, identifying the needs of public health systems and communicating them adequately to product developers should avoid the latter investing efforts and resources in solutions and products lacking adequate value. Building constructive and continuous dialogue with policymakers and public health actors involving funders, researchers, and developers, arranging workshops, seminars, and confrontation at conferences, can optimise efforts and resources towards public health goals.

#### Priority 6: urge stakeholders to have a holistic and long-term perspective on the budget when placing priorities on innovation

Innovations brought by PM benefit the prevention, prediction, and treatment of a number of diseases, bringing significant value to them. Nonetheless, the fact that R&I require long-term investments and efforts while often lacking returns in the short term could be discouraging to innovators. Finding solutions that would lower the negative short-term impact on the budget (e.g., financial incentives, such as tax deductions) would compensate for the lack of short-term benefits and incentivise innovation by recognising its added value in the long term.

#### Priority 7: elaborate on cost-effectiveness and economic analysis models that consider the social and healthcare budgets

Innovations in citizens’ education and patient empowerment only show up as costs in the social budget, but their benefits will directly translate into gains in the healthcare budget through healthier individual lifestyles and therefore a lower probability of suffering from certain diseases. Having both budgets simultaneously in focus can yield a more complete perspective on innovations and help assess their cost-effectiveness better.

#### Priority 8: extend the role of personalised diagnostics in sustainable medical treatment

Tailoring medical treatment to individual patient profiles is the prime objective of implementing PM in healthcare systems. This would allow for choosing the best treatment for the right patient at the right moment, increasing the health benefits to the patient and removing harms deriving from unnecessary diagnostic procedures and therapeutic interventions. Advancing research in PM to enhance diagnostic accuracy is crucial. This includes developing and refining diagnostic methods to optimize resource utilization for prevention and elevate healthcare value, e.g., by means of increased investment in research and policy support advocacy.

#### Priority 9: there is a greater need for early, intense, coordinated, and continued crosstalk among all PM stakeholders to support breakthrough innovations

During workshop discussions, it was emphasized that the successful transition of research and development (R&D) investments into market innovations heavily relies on the active engagement of research and innovation (R&I) stakeholders. Particularly, involving actors from the corporate sector significantly increases the likelihood of transforming investments into market-ready innovations. For PM to advance, a continuous and robust dialogue among stakeholders is essential to support ground-breaking innovations. The European framework program for research and technological development, Horizon Europe, is actively pursuing this approach. A prominent illustration of this strategy is the European Innovation Council (EIC), which targets start-ups and Small Medium Enterprises (SMEs) within the private sector, providing support for innovations with the potential for breakthroughs and disruptive impact, aiming for scalable implementation.

#### Priority 10: reward innovations that aim for a higher therapeutic value and guide innovation efforts towards the most relevant areas

HTA often depends on the priorities of the national states, maintaining a balance between cost-effectiveness and therapeutic value of medical innovation rather than following universal principles to assess money allocation correctly. By contrast, funding programmes should encourage the pursuit of innovation in fields with a high therapeutic value for patients and citizens, guiding innovation efforts towards the most relevant areas by means of increased and continuous communication aimed at addressing unmet needs and rewarding those who follow the directions given.

#### Priority 11: develop methodologies to measure the social value of pharmaceutical products and systematically use such methods, e.g., in the context of health technology assessment

The benefit deriving from the implementation of specific pharmaceutical interventions should not be only quantified economically, as this does not consider other factors deriving from the complex environment and their numerous consequences on patients. On the contrary, HTA and other evaluation methodologies should keep into account non-monetary factors, including a wide range of social, psychological, and ethical aspects, among others.

#### Priority 12: keep into account the interconnection and mutual dependency between diagnostics and therapeutics

R&I actors from the diagnostics and therapeutics fields are highly dependent on each other’s work. Nonetheless, the collaboration between different fields is often lower than needed, leading to missed opportunities and slower improvement in fields where they come together. This close interdependence between diagnostics and therapeutics is also reflected in the most recent updates in coding for healthcare reimbursement proposed by international bodies such as the World Health Organization (WHO): the current trend is to favour reimbursement systems aimed at interventions identified in their entirety over individual diagnostic or therapeutic services. In this sense, the shift toward this more holistic and inclusive view of health interventions could play a favourable role in including PM tools in reimbursement systems.

## Discussion

The fields considered by this position paper are broad.

Data management, including data exchange, global data standards, data privacy, security, and trust, is crucial to effectively manage and use data in the healthcare sector, especially within the transition to PM. In particular, the emerged priorities highlight the importance of standardising data exchange and implementing global data standards to facilitate efficient and accurate sharing of information across borders and organisations, primarily to facilitate their use for secondary purposes. This issue is currently addressed in the European Union (EU) and China, with policies and guiding opinions regulating health and medical Big Data use [3].

As PM relies on the ability to store, merge, and access large amounts of data without compromising privacy and safety, recent policies have been dedicated to addressing these concerns [21]. In the EU, emphasis was placed on ensuring data FAIRness (Findability, Accessibility, Interoperability, and Reusability), providing a European Interoperability Framework for electronic health records (EHRs) and digital health applications within and between member states, and establishing a single data market [22]. This will result in the creation of a common European Health Data Space to address health-specific challenges to electronic health data access and sharing, to be accessed by natural persons, researchers, clinicians and policy makers to support use of health data for better healthcare delivery, research, innovation, and policy making, as well as to make health and genetic data availability in electronic format homogeneous to different Member States [23]. Meanwhile, China's Guiding Opinions on Promoting and Regulating the Development of Big Data Applications for Health Care Policy in 2016 set clear rules for data governance, including the creation of a national platform for collecting, organizing, and storing health data at national, provincial, municipal, and county levels, as well as integrating it with other data sources and removing any barriers [3, 24].

The use of data in healthcare has improved and changed over time: more data is being captured and analysed than ever before, and researchers are now capturing substantially more data relative to the phenomenon they are studying. Collecting data comprehensively offers an improvement in information and using more data routinely is better than using less. Alongside with national disease registries, medical researchers will collect and analyse more comprehensive data from the population, allowing studies on the entire population rather than on samples [25]. Furthermore, enhancing standards for data availability can result in more significant benefits. Considering the imminent enactment or amendment of sectoral legal instruments pertaining to health-related data, as well as the adoption of self-regulations and policies that take into account international guidelines, the regulation of cross-border sharing of genomic data in China will be supported by more precise standards. However, it is likely to remain compartmentalised and multi-layered [24].

Both the EU and China have recognised the importance of protecting the data collected by applying a strict legal framework. In the EU, the General Data Protection Regulation (GDPR) [26] represents the most advanced legislation on EU data regulation, processing, and free movement to date, it became applicable across all EU Member States on May 25, 2018 and classifies data concerning health in Recital 3522 as personal data related to the physical or mental health of a data subject including the provision of health care services; additional principles regarding health data are detailed within the Council conclusions on Health in the Digital Society 2017/C 440/05 [27]. More recently, the EU Data Governance Act introduced secure processing environments, that are a processor-implemented method for a secure processing environment for protecting sensitive information is provided [28].

Similarly, in China’s Personal Information Protection Law (PIPL), health data, genetic data, and biometric data afford higher levels of data protection [29]. Both the GDPR and the PIPL have extraterritorial reach, but they differ in their emphasis. The GDPR primarily considers the location of the business establishment, while the PIPL focuses on where the personal information processing occurs. While both regulations share similar definitions for general personal information and individual rights, as well as key roles and legal bases for processing personal information, the PIPL has a broader scope for what constitutes "sensitive" personal information compared to the GDPR's "special category" data. It also excludes anonymous information from the definition of personal information. Additionally, the PIPL requires specific consent for processing personal information in certain situations, such as sensitive personal information or cross-border data transfer activities.

There are significant disparities in the assessment of cross-border data transfer requirements. Unlike the GDPR, which has relatively few limitations on data flow and transfer, China imposes stricter requirements under the PIPL, as well as other laws like the Cybersecurity Law and the Data Security Law. For instance, under these laws, personal information collected by “Critical Information Infrastructure Operators” must be stored within China unless a national security assessment is passed. Furthermore, regulations such as the "Measures for the Security Assessment of Outbound Data (Exposure Draft)" and the "Cybersecurity Review Measures" specify thresholds for the amount of personal information that can be transferred before a security assessment is necessary.

While these laws and regulations may initially suggest that cross-border data transfer is challenging in China, the PIPL allows it under certain conditions, pending further clarification from authorities. These conditions include passing a security assessment organized by the Cyberspace Administration of China (CAC), certification by a specialized agency for PI protection according to CAC provisions, and entering into a contract with overseas recipients using a standard contract template provided by the CAC [29]. The absence of a universally adopted and consistently executed medical terminology system poses a significant obstacle to utilizing big data in medical research. This issue was addressed during the workshops, and many related concerns emerged: for instance, although the National Health and Family Planning Commission mandated the use of the International Classification of Diseases (ICD-9, and more recently ICD-10) for all hospital patients since 2002, the proliferation of hospital information systems has resulted in various inconsistencies in the coding of other clinical terms beyond diagnosis and procedures, leading to challenges in data exchange and reimbursement in countries where the DRG system is in place. In China, widely acknowledged terminology systems like the Systematized Nomenclature of Medicine–Clinical Terms (SNOMED CT) [30], the Unified Medical Language System (UMLS) [31], or the General Architecture for Languages, Encyclopaedias, and Nomenclatures in Medicine (GALEN) [32] are not accessible. By incorporating and distributing key terminology, classification, and coding standards in medicine, these systems can promote more effective and interoperable biomedical information systems and services, including electronic health records. However, additional efforts are required to bridge the linguistic disparities between Chinese and English beyond the current translation of terms [33]. To facilitate collaboration and foster initiatives between the EU and China, it is imperative to acknowledge and tackle the linguistic disparities that exist between the Chinese and English languages. Specifically, attention must be given to the challenges associated with various Chinese dialects and regions where classical Chinese writing is still prevalent. These factors introduce notable deviations from standard Chinese, consequently intensifying the complexity of bridging language barriers. Thus, it becomes essential to address these distinctive linguistic challenges as a crucial stride towards nurturing effective communication and fostering harmonious cooperation. This involves moving beyond the mere translation of terms towards establishing a common lexicon, classification, and coding standards [34].

The identified priorities also address the importance of innovation incentives and the factors underpinning market uptake and adoption. These factors might include consumer demand, regulatory requirements, or the availability of supporting infrastructure. By understanding these factors, innovators can better position their products for market success. However, developing innovative products can be expensive, thus it is essential to understand the potential return on investment before committing significant resources. Need assessment should be thoroughly carried out in the economic evaluation.

A major challenge for the future is to integrate genetic data with other types of information, including lifestyle and environmental factors and their influence on genes and biohumoral data, within a holistic approach that can improve the traditional practice of symptom-based medicine. The study of the exposome, that is, the relationships between a person and the environment, can enable earlier interventions and personalized treatments using advanced diagnostics [34].

The identified priorities constitute a framework for addressing future efforts on the causes of suboptimal collaboration and planning additional initiatives to develop strategic actions. PM can improve patients’ quality of life by facilitating diagnosis, therapy, and care while reducing long-term costs associated with inappropriate diagnoses or ineffective therapies within the healthcare system [35]. Adopting this approach will make it feasible to enhance budgetary oversight and allocate essential resources towards the relevant domain [35]. To foster and promote collaboration in PM regarding Big Data and ICT solutions, European and Chinese policymakers and stakeholders can support breakthrough innovations and develop solutions by collaborating and sharing information and resources. They can work together to create policies that promote research and development in PM, provide funding for research, and support the development of innovative technologies and tools. It would be possible to ensure that policies and regulations are aligned with the needs and concerns of patients, healthcare providers, and the broader public. This can involve engaging with patient advocacy groups, industry associations, and academic institutions to understand their perspectives and needs.

The results of this work are to be considered in the light of strengths and limitations. The aim of this work was to identify priorities for action to create common ground for policy development, and to identify needs and new perspectives. The rigorous methodology of the project, based on extensive literature research, workshops, international comparisons and constant dialogue between the parties aims to fill a gap in the scientific literature on the most relevant areas to be addressed in PM. Therefore, this work provides, in our opinion, important food for thought and action for policymakers and the health care world, through an international perspective of collaboration and support.

Nevertheless, it is possible that the described priorities are not exhaustive of the subject matter, probably due to the lack of key documents during the search phase or due to the cultural and working background of the participants and experts. Furthermore, it should be emphasised that the project looks at the European and Chinese perspective, so the results may not be representative for other countries. In addition, we supported the goodness-of-fit of the EU-China pairing by exploring the topic in an introductory meeting with partners from both countries, trying to highlight the state of the art of PM in the two countries and the policy, social, and health frameworks in order to make the conclusions valid for both sides. However, it is possible that differences between the two countries influenced the results, and we did not take these potential issues into account in the analysis of the Delphi results. It is also possible that some priorities are more relevant in the European context and others in the Chinese context, suggesting a nation-specific scale of importance. However, this aspect, discussed during the project, was considered unimportant considering the broad scope of the priorities presented.

Furthermore, we adopted a perspective that is technology-agnostic, meaning it doesn't rely on or prioritize specific technologies. This approach allows for flexibility in deployment based on specific needs rather than being tied to particular tools or platforms. By adopting a technology-agnostic perspective, we could focus more on solving problems or addressing requirements without being constrained by the limitations or dependencies of particular technologies. This flexibility is particularly valuable in rapidly evolving fields where new technologies may emerge, or where the best solution may vary based on the context. These aspects were particularly valuable in taking into account the different expertise on the PM scenario brought by the experts involved and the different legislative and socio-cultural context in Europe and China.

## Conclusions

The priorities in this Position Paper aim to serve as a guide for decision-makers in the healthcare sector to maximise value creation to better implement ICT and Big Data solutions in the health systems. Although not comprehensive of all the criticalities that should be addressed, the literature mapping exercise and the following expert consultation have provided some of the most pressing issues. Despite not pretending to encompass all possible aspects, this paper should be regarded as an effort to assist policymakers in their decision-making process and encourage healthcare professionals, industry leaders, and researchers to join their efforts for shared profit and mutual benefit.

## Data Availability

All data generated or analysed during this study are included in this published article and available on reasonable request.

## Abbreviations

AI: Artificial Intelligence
ATMP: Advanced Therapy Medicinal Products
CROMs: Clinician Reported Outcome Measures
CVI: Codex Valid Index
EHRs: Health records
EIC: European Innovation Council
EU: European Union
FAIRness: Findability, Accessibility, Interoperability, and Reusability
GALEN: General Architecture for Languages, Encyclopaedias, and Nomenclatures in Medicine
GDPR: General Data Protection Regulation
HTA: Health Technology Assessment
ICT: Information and Communication Technologies
PIPL: Personal Information Protection Law
PM: Personalized Medicine
PREMs: Patient Reported Experience Measures
PROMs: Patient Reported Outcome Measures
R&I: Research and Innovation
SMEs: Small Medium Enterprises
SNOMED CT: Systematized Nomenclature of Medicine–Clinical Terms
UMLS: Unified Medical Language System
WHO: World Health Organization
